# From Cheese Whey Permeate to Sakacin A: A Circular Economy Approach for the Food-Grade Biotechnological Production of an Anti-*Listeria* Bacteriocin

**DOI:** 10.3390/biom10040597

**Published:** 2020-04-12

**Authors:** Alida Musatti, Daniele Cavicchioli, Chiara Mapelli, Danilo Bertoni, Johannes A. Hogenboom, Luisa Pellegrino, Manuela Rollini

**Affiliations:** 1 DeFENS, Department of Food, Environmental and Nutritional Sciences (DeFENS), Università degli Studi di Milano, Via Mangiagalli 25, 20133 Milano, Italy; chiara.mapelli1@gmail.com (C.M.); john.hogenboom@unimi.it (J.A.H.); luisa.pellegrino@unimi.it (L.P.); manuela.rollini@unimi.it (M.R.); 2 ESP, Department of Environmental Science and Policy, Università degli Studi di Milano, Via G. Celoria 2, 20133 Milano, Italy; daniele.cavicchioli@unimi.it (D.C.); danilo.bertoni@unimi.it (D.B.)

**Keywords:** cheese whey permeate, sakacin A, bacteriocin, *Listeria*, bioprocess, circular economy, fermentation

## Abstract

Cheese Whey Permeate (CWP) is the by-product of whey ultrafiltration for protein recovery. It is highly perishable with substantial disposal costs and has serious environmental impact. The aim of the present study was to develop a novel and cheap CWP-based culture medium for *Lactobacillus sakei* to produce the food-grade sakacin A, a bacteriocin exhibiting a specific antilisterial activity. Growth conditions, nutrient supplementation and bacteriocin yield were optimized through an experimental design in which the standard medium de Man, Rogosa and Sharpe (MRS) was taken as benchmark. The most convenient formulation was liquid CWP supplemented with meat extract (4 g/L) and yeast extract (8 g/L). Although, arginine (0.5 g/L) among free amino acids was depleted in all conditions, its supplementation did not increase process yield. The results demonstrate the feasibility of producing sakacin A from CWP. Cost of the novel medium was 1.53 €/L and that of obtaining sakacin A 5.67 €/10^6^ AU, with a significant 70% reduction compared to the corresponding costs with MRS (5.40 €/L, 18.00 €/10^6^ AU). Taking into account that the limited use of bacteriocins for food application is mainly due to the high production cost, the obtained reduction may contribute to widening the range of applications of sakacin A as antilisterial agent.

## 1. Introduction

The new concept of healthy food is boosting the use of natural compounds as alternatives to chemical preservatives in preventing bacterial deterioration [1]. Bio-preservation exploit non-pathogenic microorganisms and their metabolites having antimicrobial effect [2]; in this frame, bacteriocins can be good candidate compounds as long as they lack toxic effects to humans [3,4]. Chemically, bacteriocins are small proteins or peptides, heat-stable, active at acidic pH and, owing to the low concentrations required, do not modify food flavour [3,5]. Numerous classifications have been proposed based on their structure, mode of action, biochemical properties and post-translational odifications [6], nevertheless, class I and II bacteriocins are the most studied. Their targets are mainly foodborne pathogens such as *Listeria monocytogenes*, *Staphylococcus aureus*, *Escherichia coli* and *Salmonella* spp. [5]. The main obstacle to widespread use of bacteriocins in food biopreservation is the high price of their commercial preparations due to the low fermentation yields and high production costs. Complex growth media are needed, which often cannot be classified as food-grade. The MRS (de Man, Rogosa and Sharpe) medium is currently considered as the most suitable for promoting the growth of lactic acid bacteria (LAB). However, it is expensive and thus its use for industrial production of bacteriocins appears unfeasible. An attractive approach to producing food-grade bacteriocins is to use by-products of the agro-food industry in the formulation of culture media [3]. In this frame, cheese whey (CW) and whey fermentations by LAB.

CW is obtained as a by-product of cheese manufacture. It represents around 90% of the initial milk volume and contains soluble components, including lactose, whey proteins, peptides and amino acids, minerals and water-soluble vitamins. Owing to their distinctive nutritional and functional characteristics, whey proteins are mainly recovered from CW by ultrafiltration (UF) and become ingredients for a variety of foods, pharmaceuticals and cosmetics [7]. The UF process generates a further by-product, namely cheese whey permeate (CWP), that is highly perishable, has high disposal costs and serious environmental impact [8,9], but at the same time, it retains valuable milk components [10]. Liquid CWP is partly destined to lactose production, but most is turned into powder [9]. As a consequence of an expected increase in demand for whey protein concentrates, the availability of CWP will grow as well [11,12,13], which may represent a resource for activating valuable circular economy activities [14]. World CWP volume (in powder) is estimated in 855,000 Metric Tonnes (MT) in 2017, with an expected increase by 4% Compound Annual Growth Rate (CAGR) over the period 2017–2027 [15]. The associated market value of world CWP production is worth in 598 Millions of Euro in 2017. Market price for CWP powder was 0.7 €/kg in 2017 and is expected to increase up to 0.731 €/kg in 2027. Note that these data refer to CWP powder cost, that incorporates the drying step; transformation in powder is performed to make CWP transportable and storable. According to various experts’ opinion, the price of liquid unprocessed CWP is in the range 1–1.5 cent €/L, including the refrigeration costs.

Therefore, using liquid or powder CWP depends on logistic evaluations, based on the proximity between UF plants and the place in which CWP is used for circular bioeconomy activities. The conversion of dairy industry effluents from waste to resources, with different options of management and valorisation, has a long stand history [16,17]. However, the increasing societal demand for more sustainable food manufacture processes has boosted the research in this field.

In particular, the use of CWP has been experimented as a substrate to obtain bioproducts having different degrees of added value, such as lactic acid [18], biopolimers [19], biofuel and biogas [20,21] as well as microalgae [22].

*Listeria monocytogenes* is a Gram-positive, facultative anaerobe, motile, non-spore forming, rod shaped bacterium that has been known as the causative agent of listeriosis, a highly fatal opportunistic foodborne infection. Listeriosis is rare, but its social and economic impact is one of the highest among foodborne diseases [23]. Sakacin A, a bacteriocin produced by *Lactobacillus sakei*, as all class IIa bacteriocins, primarily acts by permeabilizing *Listeria* membrane, dissipating (in term of seconds) transmembrane potential (ΔΨ) and pH gradient (ΔpH) through the formation of pores [6]. Other sakacins have been identified and characterized from a variety of *L. sakei* strains, most of which isolated from raw meat. Examples are sakacin M, P, 674, B and K [24]. Sakacin A presents a 100% sequence identity with the bacteriocin produced from *Lactobacillus curvatus*, curvacin A. Although these two proteins could be considered equivalent under a molecular point of view, small differences are reported in the gene cluster organization [25]. Recently, it has been highlighted that sakacin A can find application in preserving food items potentially contaminated with *Listeria monocytogenes*, such as ready-to-eat products, with the bacteriocin successfully incorporated in food packaging material [26,27,28]. It is worth noting that the market value of the antimicrobial packaging sector is currently 29.0 billion US$ [29,30], supporting the interests of the food industry in this new technology. Economic feasibility of this approach, however, strongly lies in market availability of food-grade bacteriocins at relatively low cost.

The aim of the present study was to develop a novel and cheap CWP-based food-grade culture medium for *L. sakei* to produce sakacin A. Growth conditions of the strain, nutrient supplementation and bacteriocin yield were optimized. Liquid CWP was used as the basic ingredient in view of establishing a bioprocess that can be easily scaled up, avoiding CWP concentration and drying costs.

## 2. Materials and Methods

### 2.1. Microorganisms and Maintenance

*Lactobacillus sakei* DSMZ 6333 (Lb706) (DSMZ: Deutsche Sammlung von Mikroorganismen und Zellkulturen GmbH, Braunschweig, Germany) was the sakacin-A-producing strain while *Listeria innocua* DSMZ 20649 was the target strain. The two strains were maintained on MRS broth (DeMan-Rogosa-Sharpe, Merck K GaA, Darmstadt, Germany) and on TSB (Tryptic Soy Broth; Merck K GaA) inoculating media (10% *v*/*v*) respectively. A pre-grown culturing and incubation in stationary condition for 16 h were carried out at 30 °C for *L. sakei* and 37 °C for *L. innocua*. Stock cultures of both microorganisms were stored at −80 °C in the medium added with 20% (*v*/*v*) glycerol (VWR International, Leuven, Belgium). Cultures were propagated twice before use.

### 2.2. Quantification of Sakacin a Activity

Antimicrobial activity of sakacin A was evaluated by the agar diffusion assay, as reported in Mapelli et al. [29]. Briefly, aliquots of overnight *Listeria* culture were added to soft TSA (TSB added with 8 g/L agar) in a Petri dish, then cell-free supernatant of *L. sakei* culture was properly diluted and poured in wells previously made on the plate. Plates were incubated overnight at 37 °C and sakacin A production was quantified as Activity Units per medium (AU/mL) according to Trinetta et al. [30] or, alternatively, as the measure (mm) of inhibition halo around the wells.

### 2.3. Lactobacillus Sakei Growth Evaluation

A culture medium for *L. sakei* (SAK-medium) was initially used, having the following composition: meat extract, ME (VWR) 8 g/L; yeast extract, YE (Costantino srl, Turin, Italy) 8 g/L; arginine, ARG (Merck K GaA) 0.5 g/L; Tween-80 (Merck K GaA) 0.5 mL/L, minerals and vitamins mix 1 mL/L as reported by Mapelli et al. [27]. Ingredients were dissolved in liquid CWP, kindly supplied by Latteria Soresina (Soresina, Italy).

Freshly produced CWP was collected at the cheese factory from the UF plant that processes whey from hard cheese manufacture, immediately frozen and kept at −20 °C until use. The CWP batch had a lactose content of 42 g/L and pH 6.2 and was filter sterilized just before use.

Both *L. sakei* growth and sakacin A production were determined in the SAK-medium and in MRS, being the latter taken as benchmark. *L. sakei* growth was studied at 26°C with different percentage of inoculum, i.e., 0.1, 0.5, 1 and 5% (*v*/*v*). Culture turbidity (OD) was measured spectrophotometrically at 600 nm every 15 min in a PowerWave™ XS2 Microplate Spectrophotometer (BioTek, Winooski, VT, USA); lag phase (min) and maximum growth rate (OD/h) were determined fitting data through the Baranyi and Roberts model [31] on DMFit 3.5 Excel add-in.

### 2.4. Sakacin-A Production and Process Optimization

A general full factorial design with 3 variables was set-up to identify components and/or interactions having significant effect on sakacin A production; the chosen levels were as follows: A-ME 0–4–8 g/L; B-YE 0–4–8 g/L; C-ARG 0–0.5–1 g/L; these concentrations were chosen in order to maximize production of sakacin A while possibly reducing the cost of the culture medium. The 27 combinations were tested in duplicate, giving rise to a total of 54 trials. Culture formulations were all inoculated with a pre-grown *L. sakei* liquid culture and incubated at 26 °C to promote bacteriocin production.

Multiple factors interactions were determined employing Design Expert^®^ v. 12.0 software (Stat-Ease Inc., Minneapolis, MN, USA), combined with the Student t test to evaluate whether given terms had significant effect (*p* ≤ 0.05). Results were monitored in terms of pH value, measured with a pH-meter (FiveEasy™, Mettler Toledo, Schwerzenbach, Swiss), lactic acid production, amino acid consumption, as well as bacteriocin production, measured in terms of diameter (mm) of the inhibition halo. Quality of fitted model equations was expressed by the regression coefficient R-square. Statistical parameters and interactions significance were verified by the analysis of variance (ANOVA). Parametric estimations, calculated from the results, were carried out by minimizing the sum of quadratic differences between observed and model-predicted values, using the non-linear least-squares procedure.

### 2.5. Lactic Acid and Amino Acids Determinations

Lactic acid was determined in the culture media after centrifugation and filtration of the supernatant. An HPLC system (Merck-Hitachi L-7000 System) was used, equipped with an UV detector (210 nm), using a (300 × 8 mm) SH1821 column (Shodex, München, Germany), maintained at 50 °C and eluted with 5 mM H_2_SO_4_ at 0.5 mL/min.

Free amino acids, including added arginine, were analysed in culture sample supernatants that were further adjusted to pH 2.2 and filtered. A Biochrom 30+ automatic amino acid analyser (Biochrom Ltd., Cambridge, UK) was used and elution conditions recommended by the manufacturer were adopted. A 14-step elution program with six lithium citrate buffers of increasing pH and ionic strength was adopted, with post-column derivatization with ninhydrin and detection at 440 and 570 nm. Five-level calibration curves were used for amino acid quantification [32].

## 3. Results and Discussion

### 3.1. L. Sakei Growth and Sakacin a Production in MRS and SAK Media

The optimized process for sakacin A production, using CWP as a substrate, refers to a lab-scale cost minimization consideration. Process optimization, based on lab-scale cost minimization, may present some limitations. First, lab-scale production costs include only the cost of ingredients for medium formulation. Given the small production scale, energy, labor and fixed costs associated with lab facilities, are not included in the computation. Such omitted costs, may lead to an increase in the total costs when production is up-scaled from lab-level to pilot- and plant-level. On the other hand, up scaling the production usually brings to a decrease in average cost of production, for the well-known phenomenon of economies of scale. Such relationship is due to both, technological and market reasons. The technological reasons are explained by the increasing return to scale: an increase of 1% in production inputs (labour, ingredients and machinery/plants) brings to a production increase bigger than 1%. Market reasons that contributes to economies of scale, relate to the decreasing procurement costs of variable inputs: Growth of production increases the demand of ingredients for the medium, which in turn reduces their buying price for the plant. All the above mentioned explanations point out that lab-scale production costs are not necessarily a good forecast of plant-level production costs, even if, usually, the former are higher of the latter. Another limitation of optimization, based on lab-scale production costs, is the comparability across processes in different market conditions. The production cost is computed by multiplying the quantity of each ingredient used in 1 L of medium, per unit cost (price). For this reason, production cost of processes presented in this paper are comparable among them and with other process of production of sakacin A only under the assumption of equal price (per-unit cost) of the ingredients used in the medium. Nevertheless, to ensure comparability among production processes in this paper and from other authors, we provide the composition of each medium/formulation, so that, for each process, the production cost can be recomputed based on different market prices of each ingredient.

The initial formulation of SAK-medium, the prices of all ingredients used in the present analysis as well as that of MRS, taken on the basis of their cost at lab scale, are shown in Table 1. Meat extract (ME), yeast extract (YE) and Tween 80 are also present in MRS, the first two to support microbial growth, while Tween 80 reduces the mean particle size and polydispersity index of ingredients, thus, allowing a fastest microbial uptake [33]. All minerals present in MRS (sodium acetate, MgSO_4_, MnSO_4_, ammonium citrate, potassium phosphate) were avoided and replaced by those naturally present in CWP. The initial concentration of ME, YE and the other ingredients, dissolved in CWP, have been chosen on the basis of previously published data [26,27]. The presence of arginine was evaluated to investigate the possibility of promoting the arginine deiminase (ADI) pathway, adopted by a variety of bacterial species for obtaining further ATP for cell growth and NH_3_ that helps in maintaining the pH to physiological levels. The enzymes involved in the ADI pathway catalyze the conversion of arginine to citrulline, NH_3_ and CO_2_, while generating ATP. Citrulline can be further metabolized into ornithine and carbamoyl-phosphate [34].

Based on this composition, the SAK-medium was cheaper (−58%) than MRS, i.e., 2.30 and 5.40 €/L, respectively. Furthermore, the cost share of CWP on total cost of SAK was the lowest among ingredients, confirming CWP as a potential cheap substrate.

**Figure 1:**
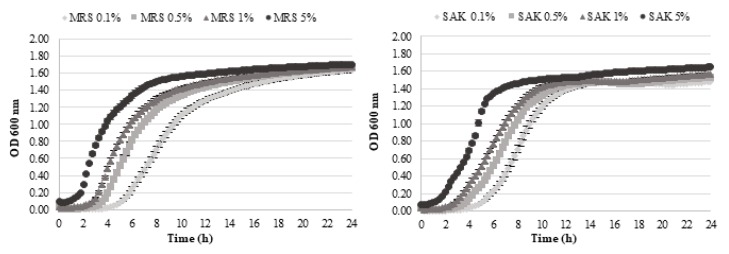
*L. sakei* DSM 6333 growth in MRS (left) and SAK (right) formulation at 26 °C. Different curves correspond to different percentage of inoculum (*v*/*v*), from 0.1% (light grey) to 5% (black).

*L. sakei* growth was monitored comparatively in SAK and MRS media differently inoculated (0.1–0.5–1–5% *v*/*v*) (Figure 1). Analyses of the fitted growth curves identified *L. sakei* lag phase (h), growth rate (OD/h) and final OD value (Table 2).

As expected, the lag phase significantly decreased with the increase of inoculum percentage for both media and, regardless to the inoculum, longer in SAK. Even if slightly higher *L. sakei* growth (final OD) was obtained in MRS, growth rates were always higher in SAK, highlighting that the formulation containing CWP can effectively support *L. sakei* growth. The quantification of the antimicrobial activity confirmed that the highest sakacin A production was reached at the end of the exponential phase of each curve [27,35], i.e., at 8 h for cultures inoculated at 5% (*v*/*v*) (300 and 333 AU/mL in MRS and SAK respectively) and 16 h for 0.1–0.5% (*v*/*v*) (233–267 AU/mL).

### 3.2. Optimization of SAK Medium Formulation

Different formulations of the initial SAK medium were tested with the possibility of reducing their amount, in order to further reduce sakacin A production costs. Ingredients were evaluated for their influence on pH decrease, lactic acid and sakacin A production (Table 3). As previously explained, pH time course cannot be considered totally representative of sakacin A production due to the possible activation of the ADI pathway with consequent NH_3_ production. On the contrary, as lactic acid production is directly linked to bacterial growth, its concentration can be correlated to the presence of sakacin A [27]; concentrations of lactic acid higher than 2 g/L were found when YE concentration was set at its maximum level and above all when in presence of ME, while arginine seems not to be correlated with microbial growth and lactic acid production.

**Figure 2:**
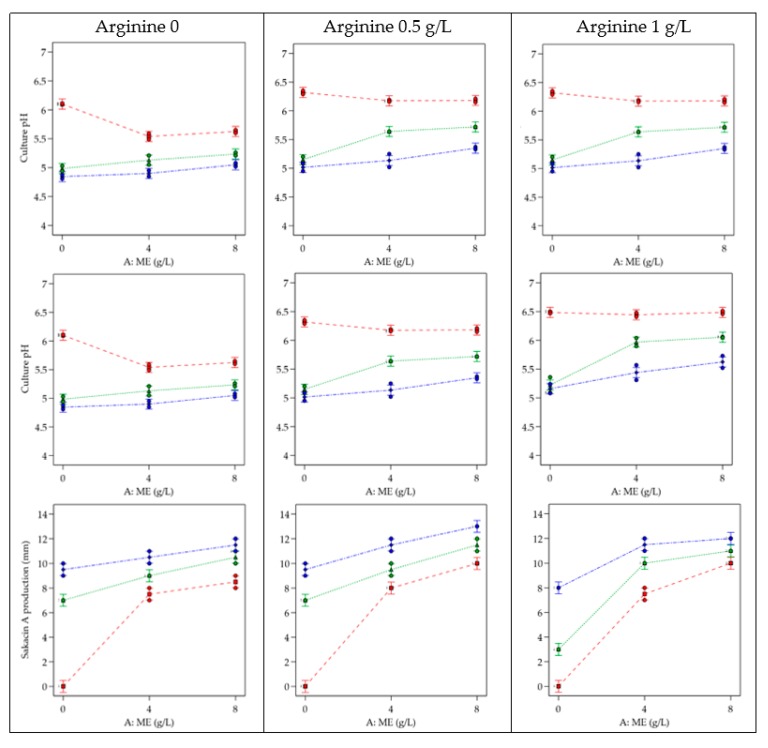
Interactions plots: responses related to culture pH (first row), lactic acid (second row) and sakacin A production (third row, expressed as inhibition halo vs. *Listeria*) in supernatants of *L. sakei*. Factors: A-ME (Meat Extract) at 0-4-8 g/L on X-axis; B-YE (Yeast Extract) at 0 g/L (red)–4 g/L (green)–8 g/L (blue); results obtained without arginine (first column), with arginine set at 0.5 g/L (second column) and 1 g/L (third column).

ANOVA analyses identified as significant positive terms A-ME, B-YE and the combination AB (Table 4); as expected, in pH responses as expected the term C-Arg and the interactions AC and BC were found significant; as for sakacin A production, all the terms and interactions resulted significant. Model adaptation goodness, variability and noise ratio were found adequate for all the three models identified by the software. Figure 2 reports the interaction plots obtained with observed-predicted pH values, lactic acid concentration levels and sakacin A production obtained in trials performed without (i) or with 0.5 g/L (ii) and 1 g/L (iii) arginine.

The lowest pH levels were always associated with the highest YE concentration (blue line), while a low influence of ME was observed. As expected, higher pH levels were evidenced in trials with arginine, confirming the activation of the ADI pathway.

Both, lactic acid and sakacin A productions, were at their lowest levels when no YE and ME were added, i.e., when microbial growth was limited. A direct correlation was evident between the concentration of these two ingredients and lactic acid production, YE being more influent than ME on *L. sakei* growth and bacteriocin production. This ingredient, due to the presence of amino acids and peptides, has already been reported to promote LAB cell growth [4].

The analysis of free amino acids was also performed (Table 5): Trials containing 0.5 g/L arginine (Table 3, Run 9) and the correspondent, without (Table 3, Run 27) evidence that arginine was totally depleted in both samples, as expected, and ornithine steeply increased. Cysteine and glutamine were absent either at t0 and at the end of incubation in both the formulation without or with arginine.

Serine was found to sharply decrease during cell growth of about −98, and −85%, respectively in samples without and with arginine. Serine decrease during *L. sakei* growth may indicate the possible existence of metabolic routes aimed to produce energy; serine conversion into pyruvate has been described in *P. pentosaceus* due to the action of a serine dehydratase [36].

McLeod et al. [37] observed a drastic utilization of serine and asparagine in *L. sakei* strains grown under glucose limiting conditions. Montanari et al. [38] calculated a 30% decrease of the serine + asparagine concentration in *L. sakei* cultured in MRS medium; here, a 55% decrease of this amino acid association was evidenced with no significant difference related to the presence of arginine.

The optimization approach suggested by the software (desirability higher than 0.800) to maximize sakacin A and lactic acid production while minimizing pH, resulted in 7 medium formulations, reported in Table 6 together with their respective formulation cost (€/L), sakacin A production yield (AU/L) and production cost (€/10^6^ AU).

Keeping in mind the final goal of the present work, the most convenient SAK formulation was found composed by ME at 4 g/L, YE at 8 g/L and no arginine, having a formulation cost of around 1.53 €/L and an estimated sakacin A cost of around 5.67 €/10^6^ AU. Compared with the values obtained in MRS (5.40 €/L, 18.00 €/10^6^ AU), a 70% decrease in the production costs was reached in this study. Mapelli et al. [27] employing the non-optimized SAK formulation, resulted in 2.33 €/L and 8.06 €/10^6^ AU, around 30% higher. Trinetta et al. [30] reported, for sakacin A, higher production costs (2.9 €/L), 50% more expensive medium containing bacto peptone, meat peptone, cheese whey, yeast autolysate, glucose and calcium carbonate.

Even if prices are calculated on the basis of ingredients purchased in lab-scale mass, difference in cost among the selected formulations still can be compared to identify the most convenient choice. Note also, that when up-scaling the quantity to be purchased, the cost will be reduced accordingly: so far, starting from the formulation here reported, the production cost of sakacin A is likely to be lower than that reported here. Taking into account that the limited use of bacteriocins in the production of antimicrobial materials for food packaging solutions is mainly due to their high production cost, the obtained reduction may widen the range of applications of these molecules as antilisterial food-grade compounds.

Mapelli et al. [29] set-up an antilisterial packaging material, based on sakacin A incorporated in cellulose nano-fibers (CNF), and demonstrated its antimicrobial activity on smoked salmon fillets intentionally inoculated with *Listeria*. The active material was prepared at a sakacin A concentration of around 50 AU/cm^2^ employing a culture medium with a formulation cost of around 8.06 €/10^6^ AU; at the reported conditions, production cost of the active mat in terms of sakacin A content resulted around 4.03 €/m^2^. With the here proposed new culture medium formulated with liquid CWP, bacteriocin production cost can be reduced from 2.33 to 1.53 €/L (5.67 €/10^6^ AU), with a cost reduction of the active material up to 2.84 €/m^2^ (−30%).

## 4. Conclusions

The present paper contributes to CWP valorization by applying the circular economy concepts expressed by de la Caba et al. [29]. This required a food industry by-product to produce antimicrobials for food preservation. This residue has been used to optimize a culture medium formulation suitable for the production of sakacin A, bacteriocin with a specific antilisterial activity obtained by *Lactobacillus sakei*. The six best formulations identified as effective in maximizing sakacin A production yield, significantly differed for their bacteriocin production cost. From an economic perspective the culture medium did not always give the highest bacteriocin production titre has the cheapest formulation. The research highlighted up to 70% decrease of sakacin A production costs can be reached compared to what obtainable in the MRS reference medium, and even in the other already published papers. This reduction will pave the way for the possibility of extending the use of sakacin A as natural antimicrobial in the production of active packaging solutions to face the risk of *Listeria monocytogenes* outbreaks in food items.

## Figures and Tables

**Table 1:** Ingredients cost of MRS (benchmark) and SAK culture media (lab scale).

Medium	Ingredient	Cost €/Kg	g/L	€/L	Share of SAK Cost (%)
MRS	As purchased	104	52.2	5.40	-
SAK	Yeast Extract	60	8	0.48	21
	Meat Extract	149	8	1.19	52
	Arginine	415	0.5	0.20	9
	Tween-80	83.7	0.5	0.04	2
	Vitamin mix	0.37 €/mL	1 mL	0.37	16
	CWP	0.015 €/L	1 L	0.015	1
			TOTAL	2.30	100

**Table 2:** *L. sakei* growth curves fitting parameters in MRS and SAK media differently inoculated (from 0.1 to 5% *v*/*v*).

Inoculum Medium		0.1% (*v*/*v*) MRS SAK		0.5% (*v*/*v*) MRS SAK		1% (*v*/*v*) MRS SAK		5% (*v*/*v*) MRS SAK	
Lag phase (h)	mean	3.267 ^A^	5.266 ^B^	2.581 ^A^	3.794 ^B^	1.854 ^A^	2.895 ^B^	0.010 ^A^	1.839 ^B^
	std. dev	0.009	0.109	0.063	0.111	0.083	0.061	0.010	0.067
Growth rate (OD/h)	mean	0.200 ^A^	0.255 ^B^	0.219 ^A^	0.239 ^B^	0.237 ^A^	0.250 ^B^	0.241 ^A^	0.306 ^B^
	std. dev	0.009	0.004	0.007	0.002	0.009	0.002	0.014	0.009
Final Value (OD)	mean	1.517 ^B^	1.459 ^A^	1.550 ^B^	1.452 ^A^	1.558 ^B^	1.496 ^A^	1.613 ^B^	1.534 ^A^
	std. dev	0.031	0.013	0.005	0.063	0.016	0.018	0.019	0.005
Sakacin A	AU/mL	267	233	267	233	267	267	300	333
	Time (h)	16	16	16	16	12	12	8	8

Values with different superscripts uppercase (^A^, ^B^) letter within the same raw are significantly different (*p* < 0.05).

**Table 3:** Arrangement of the experimental design performed with *L. sakei* grown at 26 °C for 16 h (ME-Meat Extract, YE-Yeast Extract, Arg-arginine) and observed responses: Culture pH, lactic acid concentration (g/L) and sakacin A production (expressed as inhibition halo-mm-vs. *L. innocua*).

Trial	A-ME (g/L)	B-YE (g/L)	C-Arg (g/L)	Culture pH		Lactic Acid (g/L)		Sakacin A Prod. (mm)	
				Mean Std. dev		Mean Std. dev.		Mean Std. dev	
*1*	0	0	0.5	6.32	0.04	0.01	0.00	0.0	0.0
*2*	0	4	0.5	5.15	0.07	1.35	0.00	7.0	0.0
*3*	0	8	0.5	5.02	0.09	1.84	0.21	9.5	0.7
*4*	4	0	0.5	6.18	0.02	0.68	0.00	8.0	0.0
*5*	4	4	0.5	5.64	0.00	1.56	0.01	9.5	0.7
*6*	4	8	0.5	5.14	0.16	2.35	0.13	11.5	0.7
*7*	8	0	0.5	6.18	0.04	0.98	0.01	10.0	0.0
*8*	8	4	0.5	5.72	0.00	1.81	0.05	11.5	0.7
*9*	8	8	0.5	5.35	0.03	2.59	0.02	13.0	0.0
*10*	0	0	1	6.49	0.02	0.01	0.00	0.0	0.0
*11*	0	4	1	5.23	0.19	1.20	0.18	3.0	0.0
*12*	0	8	1	5.16	0.11	1.70	0.33	8.0	0.0
*13*	4	0	1	6.45	0.05	0.80	0.01	7.5	0.7
*14*	4	4	1	5.97	0.10	1.57	0.05	10.0	0.0
*15*	4	8	1	5.44	0.18	2.47	0.04	11.5	0.7
*16*	8	0	1	6.49	0.04	1.03	0.06	10.0	0.0
*17*	8	4	1	6.06	0.01	1.88	0.04	11.0	0.0
*18*	8	8	1	5.63	0.15	2.66	0.13	12.0	0.0
*19*	0	0	0	6.10	0.01	0.03	0.01	0.0	0.0
*20*	0	4	0	4.99	0.06	1.33	0.10	7.0	0.0
*21*	0	8	0	4.85	0.05	1.93	0.00	9.5	0.7
*22*	4	0	0	5.54	0.07	0.69	0.06	7.5	0.7
*23*	4	4	0	5.13	0.11	1.45	0.07	9.0	0.0
*24*	4	8	0	4.90	0.07	2.11	0.26	10.5	0.7
*25*	8	0	0	5.63	0.04	0.90	0.06	8.5	0.7
*26*	8	4	0	5.24	0.04	1.74	0.06	10.5	0.7
*27*	8	8	0	5.05	0.04	2.44	0.07	11.5	0.7
MRS	-	-	-	4.45	0.10	5.62	0.18	13.0	0.7

**Table 4:** ANOVA response, effect estimates, models adaptation goodness as well as variability and noise ratio for each of the observed responses (pH, lactic acid and sakacin A production) of the experimental design reported in Table 3 (α-out: 0.05).

Parameter	pH	Lactic Acid	Sakacin A Production
Model	*p* < 0.0001 Linear	*p* < 0.0001 Square root	*p* < 0.0001 Linear
A-ME	*p* < 0.0001	*p* < 0.0001	*p* < 0.0001
B-YE	*p* < 0.0001	*p* < 0.0001	*p* < 0.0001
C-Arg	*p* < 0.0001	ns *	*p* < 0.0001
AB	*p* < 0.0001	*p* < 0.0001	*p* < 0.0001
AC	*p* < 0.0001	ns	*p* < 0.0001
BC	*p* 0.0093	ns	*p* 0.0040
ABC	ns	ns	*p* 0.0008
Lack of fit	0.457 ns	0.1066 ns	- (Saturated)
Model std. dev	0.086	0.048	0.047
mean	5.59	1.13	0.85
C.V.%	1.53	4.25	5.56
R^2^	0.9828	0.9894	0.9916
Adj R^2^	0.9740	0.9875	0.9834
Pred R^2^	0.9591	0.9847	0.9663
Adeq precision	32.449	75.839	39.000

* ns: not significant, *p* value > 0.0500.

**Table 5:** Free amino acid concentration (mg/L) at t0 and 16 h incubation and percent variation in *L. sakei* grown cultures in SAK medium without or with L-arginine addition (0.5 g/L). Data reported as means, CV in the range 7–12%.

Amino Acid	YE 8-ME 8-ARG 0			YE 8-ME 8-ARG 0.5		
(mg/L)	t0	16 h	(%)	t0	16 h	(%)
Alanine	171.0	183.8	7	169.3	183.4	8
Arginine	194.0	0.0	−100	526.0	0.0	−100
Asparagine	78.0	63.6	−18	76.5	56.7	−26
Aspartate	138.4	127.8	−8	137.5	131.9	−4
Citrulline	26.5	24.1	−9	25.8	24.9	−3
Cysteine	0.0	0.0	0	0.0	0.0	0
GABA	10.2	10.4	2	10.1	10.5	4
Glutamine	0.0	0.0	0	0.0	0.0	0
Glutamate	402.6	429.9	7	392.3	423.6	8
Glycine	70.0	89.0	27	69.1	89.9	30
Histidine	44.2	49.3	12	45.8	50.6	10
Isoleucine	131.7	175.4	33	129.9	175.9	35
Leucine	254.5	296.0	16	251.2	293.5	17
Lysine	189.5	216.8	14	185.7	214.9	16
Methionine	50.2	51.9	3	48.0	53.0	10
Ornithine	15.8	142.4	802	15.1	233.8	1449
Phenilalanine	180.3	201.1	12	180.6	202.4	12
Proline	52.8	70.2	33	52.0	71.3	37
Serine	98.4	11.4	−88	94.5	5.2	−95
Threonine	89.4	124.9	40	88.7	117.9	33
Tryptophan	40.1	54.1	35	39.6	54.4	37
Tyrosine	95.7	141.4	48	138.8	180.7	30
Valine	176.6	221.1	25	177.2	220.7	25

**Table 6:** Media formulations calculated by the optimization process (Design Expert^®^, desirability >0.800), maximizing lactic acid and sakacin A production: estimated cost of formulation (€/L), sakacin A production yield (10^6^ AU/L) and cost (€/10^6^ AU) reported and compared with those obtained with MRS (benchmark).

Medium	ME (g/L)	YE (g/L	Arg (g/L)	pH	Lactic Acid (g/L)	Desirability	Medium Cost (€/L)	Sak. A Yield (10^6^ AU/L)	Sak. A Cost (€/10^6^ AU)
1 (SAK)	8	8	0.5	5.35	2.56	0.907	2.30	0.289	7.96
2	8	8	0	5.09	2.56	0.907	2.13	0.242	8.80
3	4	8	0	4.84	2.30	0.878	1.53	0.270	5.67
4	4	8	0.5	5.16	2.30	0.870	1.73	0.278	6.22
5	0	8	0.5	4.98	1.82	0.825	1.14	0.190	7.37
6	4	8	1	5.47	2.30	0.823	1.93	0.233	8.28
7	8	8	1	5.65	2.56	0.819	2.53	0.233	10.86
MRS	-	-	-	4.45	5.62	-	5.40	0.300	18.00

## References

[1] Gálvez A., Abriouel H., Lucas López R., Ben Omar N. (2007). Bacteriocin-based strategies for food biopreservation. Int. J. Food Microbiol..

[2] Settanni L., Corsetti A. (2008). Application of bacteriocins in vegetable food biopreservation. Int. J. Food Microbiol..

[3] Garsa A.K., Kumariya S., Sood S.K., Kumar A., Kapila S. (2014). Bacteriocin Production and Different Strategies for Their Recovery and Purification. Probiot. Antimicrob. Prot..

[4] Sidooski T., Brandelli A., Bertoli S.L., Souza C.K., Carvalho L.F. (2019). Physical and nutritional conditions for optimized production of bacteriocins by lactic acid bacteria—A review. Crit. Rev. Food Sci. Nutr..

[5] Kaškonienė V., Stankevičius M., Bimbiraitė-Survilienė K., Naujokaitytė G., Šernienė L., Mulkytė K., Malakauskas M., Maruška A. (2017). Current state of purification, isolation and analysis of bacteriocins produced by lactic acid bacteria. Appl. Microbiol. Biotechnol..

[6] Mapelli C., Barbiroli A., De Benedetti S., Musatti A., Rollini M., Ferranti P., Berry E.M., Anderson J.R. (2018). Antilisterial bacteriocins for food security: The case of sakacin A. Encyclopaedia of Food Security and Sustainability.

[7] Ganju S., Gogate P.R. (2017). A review on approaches for efficient recovery of whey proteins from dairy industry effluents. J. Food Eng..

[8] Prazeres A.R., Carvalho F., Rivas J. (2012). Cheese whey management: A review. J. Environ. Manag..

[9] Cassano A., Conidi C., Castro-Muñoz R., Galanakis C.M. (2019). Current and Future Applications of Nanofiltration in Food Processing. Separation of Functional Molecules in Food by Membrane Technology.

[10] Zotta T., Solieri L., Iacumin L., Picozzi C., Gullo M. (2020). Valorization of cheese whey using microbial fermentations. Appl. Microbiol. Biotechnol..

[11] Lagrange V., Whitsett D., Burris C. (2015). Global market for dairy proteins. J. Food Sci..

[12] European Commission (2016). Prospects for EU Agricultural Markets and Income 2016–2026.

[13] OECD/FAO Agricultural Outlook 2019–2028.

[14] Pescuma M., de Valdez G.F., Mozzi F. (2015). Whey-derived valuable products obtained by microbial fermentation. Appl. Microbiol. Biotechnol..

[15] (2019). Permeate Market: Global Industry Analysis 2012–2016 and Opportunity Assessment 2017–2027.

[16] Korhonen H., Pihlanto-Leppälä A., Rantamäki P., Tupasela T. (1998). The functional and biological properties of whey proteins: Prospects for the development of functional foods. Agric. Food Sci..

[17] Božanić R., Barukčić I., Lisak K. (2014). Possibilities of whey utilisation. Austin J. Nutr. Food Sci..

[18] Gonzalez M.I., Alvarez S., Riera F., Alvarez R. (2007). Economic evaluation of an integrated process for lactic acid production from ultrafiltered whey. J. Food Eng..

[19] Wolf O., Crank M., Patel M., Marscheider-Weidemann F., Schleich J., Hüsing B., Angerer G. (2005). Techno-economic feasibility of large-scale production of bio-based polymers in Europe. Eur. Communities.

[20] Espinosa-Gonzalez I., Parashar A., Bressler D.C. (2014). Heterotrophic growth and lipid accumulation of Chlorella protothecoides in whey permeate, a dairy by-product stream, for biofuel production. Bioresour. Technol..

[21] Colombo B., Calvo M.V., Sciarria T.P., Scaglia B., Kizito S.S., D’Imporzano G., Adani F. (2019). Biohydrogen and polyhydroxyalkanoates (PHA) as products of a two-steps bioprocess from deproteinized dairy wastes. Waste Manag..

[22] Ende S.S., Noke A. (2019). Heterotrophic microalgae production on food waste and by-products. J. Appl. Phycol..

[23] Food and Agriculture Organization of the United Nations/World Health Organization (FAO/WHO) (2004). Risk assessment of Listeria Monocytogenes in Ready-To-Eat Foods, Technical Report. Microbiological Risk Assessment. https://apps.who.int/iris/handle/10665/42874.

[24] Delves-Broughton J., Baines D., Seal R. (2012). Natural antimicrobials as additives and ingredients for the preservation of foods and beverages. Natural Food Additives, Ingredients and Flavourings.

[25] Axelsson L., Holck A. (1995). The genes involved in production of and immunity to sakacin A, a bacteriocin from Lactobacillus sake Lb706. J. Bacteriol..

[26] Barbiroli A., Musatti A., Capretti G., Iametti S., Rollini M. (2017). Sakacin-A antimicrobial packaging to reduce Listeria contamination in thin-cut meat. J. Sci. Food Agric..

[27] Mapelli C., Musatti A., Barbiroli A., Seini S., Bras J., Cavicchioli D., Rollini M. (2019). Cellulose nano-fibers (CNF)—Sakacin-A active material: Production, characterization and application in storage trials of smoked salmon. J. Sci. Food Agric..

[28] Future Market Insights Protective Packaging Market: Global Industry Analysis and Opportunity Assessment 2015–2025. http://www.futuremarketinsights.com/reports/protective-packaging-market.

[29] de la Caba K., Guerrero P., Trung T.S., Cruz-Romero M., Kerry J.P., Fluhr J., Maurer M., Kruijssen F., Albalat A., Bunting S. (2019). From seafood waste to active seafood packaging: An emerging opportunity of the circular economy. J. Clean. Prod..

[30] Trinetta V., Rollini M., Manzoni M. (2008). Development of a low cost culture medium for sakacin A production by *L. Sakei*. Proc. Biochem..

[31] Baranyi J., Roberts T.S. (1994). A dynamic approach to predicting bacterial growth in food. Int. J. Food Microbiol..

[32] Hogenboom J.A., D’Incecco P., Fuselli F., Pellegrino L. (2017). Ion-exchange chromatographic method for the determination of the free amino acid composition of cheese and other dairy products: An inter-laboratory validation study. Food Anal. Meth..

[33] Nielsen C.K., Kjems J., Mygind T., Snabe T., Meyer R.L. (2016). Effects of Tween 80 on Growth and Biofilm Formation in Laboratory Media. Front. Microbiol..

[34] D’Incecco P., Gatti M., Hogenboom J.A., Bottari B., Rosi V., Neviani E., Pellegrino L. (2016). Lysozyme affects the microbial catabolism of free arginine in raw-milk hard cheeses. Food Microbiol..

[35] Campos C.A., Castro M.P., Rivas F.P., Schelegueda L.I., Méndez-Vilas A. (2013). Bacteriocins in food: Evaluation of the factors affecting their effectiveness. Microbial Pathogens and Strategies for Combating them: Science, Technology and Education.

[36] Irmler S., Bavan T., Oberli A., Roetschi A., Badertscher R., Guggenbühl B., Berthoud H. (2013). Catabolism of Serine by Pediococcus acidilactici and Pediococcus pentosaceus. Appl. Environ. Microbiol..

[37] McLeod A., Mosleth E.F., Rud I., Branco dos Santos F., Snipen L., Liland K.H. (2017). Effects of glucose availability in Lactobacillus sakei: Metabolic change and regulation of the proteome and transcriptome. PLoS ONE.

[38] Montanari C., Barbieri F., Magnani M., Grazia L., Gardini F., Tabanelli G. (2018). Phenotypic diversity of Lactobacillus sakei strains. Front. Microbiol..

